# Emerging evidence for a mechanistic link between low-frequency oscillation of ventricular repolarization measured from the electrocardiogram T-wave vector and arrhythmia

**DOI:** 10.1093/europace/euab009

**Published:** 2021-04-20

**Authors:** Peter Taggart, Esther Pueyo, Stefan van Duijvenboden, Bradley Porter, Martin Bishop, David A Sampedro-Puente, M Orini, B Hanson, Christopher A Rinaldi, Jaswinder S Gill, Pier Lambiase

**Affiliations:** 1 Department of Cardiovascular Sciences, University College London, London, UK; 2 BSICOS Group, 13A, 11S, Aragon, University of Zaragoza, Spain; 3 CIBER-BBN, Zaragoza, Spain; 4 Department of Imaging Sciences and Biomedical Engineering, KCL, London, UK; 5 UCL Mechanical Engineering, University College London, London, UK; 6 Cardiology Department, Guys and St Thomas‘ Hospital, London, UK

**Keywords:** Repolarization, T wave, Low-frequency oscillations, Electrophysiology, Beta-adrenergic stimulation, Action potential duration, Mechano-electric feedback, Arrhythmia

## Abstract

Strong recent clinical evidence links the presence of prominent oscillations of ventricular repolarization in the low-frequency range (0.04–0.15 Hz) to the incidence of ventricular arrhythmia and sudden death in post-MI patients and patients with ischaemic and non-ischaemic cardiomyopathy. It has been proposed that these oscillations reflect oscillations of ventricular action potential duration at the sympathetic nerve frequency. Here we review emerging evidence to support that contention and provide insight into possible underlying mechanisms for this association.

## Introduction

Oscillations of ventricular repolarization measured from the electrocardiogram (ECG) T-wave vector have recently been shown to be one of the strongest predictors of arrhythmia and sudden death in cardiac patients in a large prospective multicentre study. The results provide clear evidence that a fluctuating pattern of ventricular repolarization at a frequency <0.1 Hz, when enhanced, is highly predictive of ventricular arrhythmia and sudden cardiac death in cardiac patients.^1^ This trial builds on previous work in which the ECG T-wave vector angle was first shown to exhibit oscillations in the low-frequency (LF) spectral range (<0.1 Hz, generally one cycle in a little over 10 s). These oscillations referred to as periodic repolarization dynamics (PRD) were independent of respiration and heart rate variability and were considered to represent oscillations of ventricular repolarization.^2^ Periodic repolarization dynamics was shown to be strongly predictive of total mortality and cardiac mortality in post-MI patients^2^ and of arrhythmia risk in a retrospective analysis of data from the MADIT-2 study.^3^ A subsequent large multicentre prospective trial involving 44 centres in 15 EU countries conducted between 2014 and 2019 showed that PRD strongly predicted shocks in implantable cardioverter-defibrillator (ICD) patients and predicted mortality in conservatively treated patients.^4^

Despite the obvious importance of these findings, the link between oscillation of the ECG T-wave vector and ventricular arrhythmia is at present unclear. Understanding the electrophysiological basis for this association is important in order to refine the PRD and facilitate its use as a potential clinical tool for risk stratification. Furthermore, the link between oscillatory behaviour of repolarization and ventricular arrhythmia may provide valuable insight into arrhythmia mechanisms. In this regard, several key questions arise. What does the ECG T-wave vector angle represent? It has been proposed that these oscillations of T-wave dynamics represent the effect of phasic changes in sympathetic activation on ventricular repolarization possibly associated with changes in action potential duration (APD) related to different layers of the myocardium. But does APD exhibit oscillations? If so what drives the oscillatory behaviour? What are the electrophysiological mechanisms linking APD oscillation and ventricular arrhythmia? Do LF oscillations of ventricular repolarization interact with proarrhythmic mechanisms such as beat-to-beat variability of repolarization and T-wave alternans? Here we review emerging evidence for a mechanistic basis to help answer these questions.

## The electrocardiogram T-wave vector

**Figure 1:**
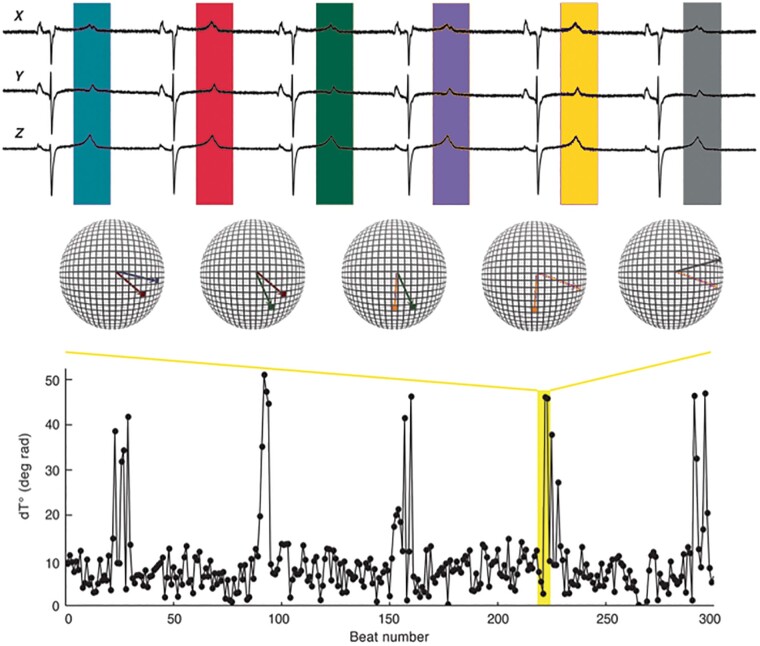
Recording of ECG T-wave vector measures of ventricular repolarization (PRD). Upper panel: ECGs are recorded using the X, Y, Z configuration. (Middle) T waves are converted into polar vector angles. Two successive T-wave vectors are shown projected onto virtual spheres. The angle between successive vectors is taken as a measure of repolarization stability. Lower panel: Plot of vector angle over time showing pronounced regular peaks with a frequency of about 0.1 Hz, i.e. 1 per 10 s. From Rizas *et al.*^2^ with permission.

**Figure 2:**
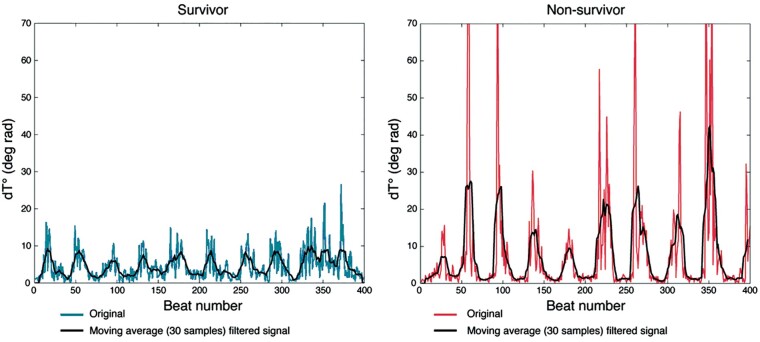
Low-frequency oscillations of the ECG T-wave vector and ventricular APD. Low-frequency ECG T-wave oscillations in a post-MI survivor (left) and a non-survivor (right) showing higher amplitude oscillations in the non-survivor. From Rizas *et al.*^2^ with permission.

The ECG T-wave vector reflects the spatiotemporal orientation of the repolarization wavefront with respect to the body surface. Oscillations of the T-wave vector referred to as PRD occur in the LF range (<0.1 Hz, *Figure 1*). In *Figure 2*, PRD recordings are shown from a post-MI patient who survived a 5-year period (left) and a patient who died 8 months after an MI (right). Typical PRD oscillations are seen which are of much greater amplitude in the patient who died compared to the survivor. It was proposed that these T-wave dynamics represent oscillations of repolarization which in turn reflect oscillations of ventricular APD.^2^^,^^4^

## Ventricular action potential duration may exhibit low-frequency oscillations

**Figure 3:**
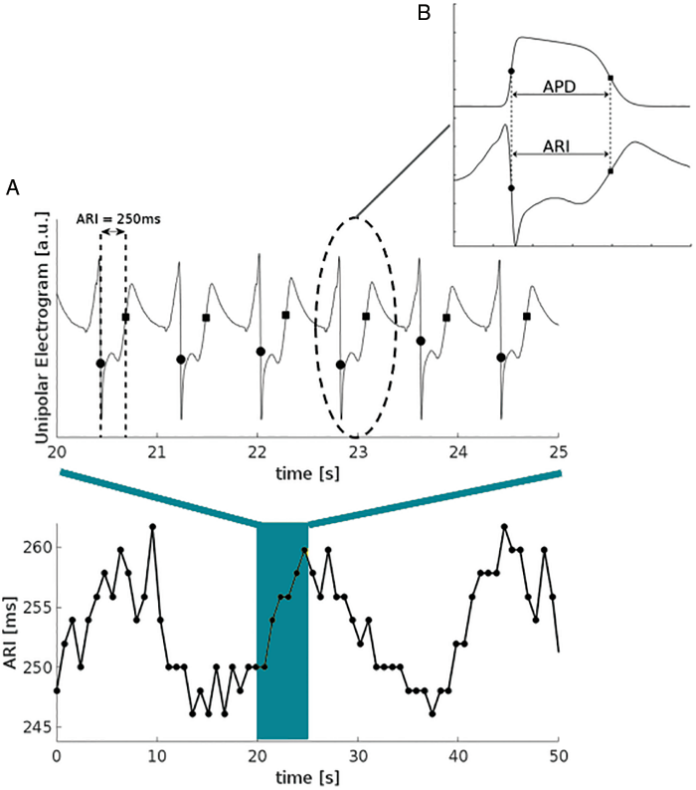
Recording of activation-recovery intervals (APD). (*A*) Upper panel: Unipolar electrogram recorded from the LV lead of an ICD device in a patient. Dots represent dv/dt min of the QRS and dv/dt max of the T wave. The interval between them defines the ARI widely used as a surrogate for APD. Lower panel: Beat-by-beat plot of ARI showing oscillatory pattern at a frequency of about 15–20 s, 0.05 Hz within the LF range. (*B*) Activation recovery interval provides an established measure of APD.

**Figure 4:**
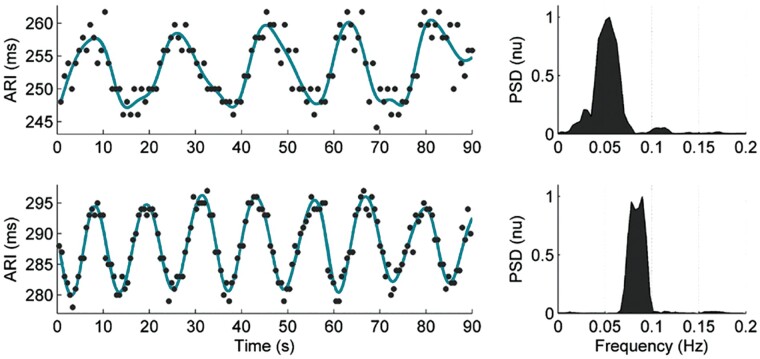
Beat-to-beat plot of left ventricular ARIs as a surrogate for APD showing oscillations in the low-frequency range at ∼0.05 Hz (upper trace) and 0.1 Hz (lower trace). Corresponding power spectral densities (PSD) are shown to the right of each trace.

Oscillatory behaviour is a ubiquitous property throughout many biological systems. However, it is only relatively recently that ventricular APD has been shown to exhibit oscillatory behaviour.^5^^,^^6^ This was first observed in patients undergoing routine electrophysiological procedures for supraventricular arrhythmias using left and right ventricular endocardial catheter electrodes. Activation recovery intervals (ARIs) derived from unipolar electrograms as a conventional surrogate for APD^7–9^ showed oscillations at the LF spectral range in the region 0.04–0.15 Hz^5^^,^^6^ (*Figure 3*). Oscillations typically occurred over a fairly narrow range within the LF spectrum (*Figure 4*). Low-frequency oscillations were subsequently observed in a number of other studies including recordings from the left and right ventricular endocardium,^10^ studies recording ARIs from the left ventricular epicardium in ambulatory patients with an implanted cardioverter-defibrillator^1^^,^^11^^,^^12^ and from monophasic action potential recordings in an established animal model.^13^

## Low-frequency oscillations of ventricular action potential duration enhanced by sympathetic provocation

The question arises as to what drives these rhythmic fluctuations of APD. It was suggested that LF oscillations of the ECG T-wave vector could be related to the characteristic oscillations of sympathetic nerve activity at this frequency.^14^ This proposal was supported by clinical studies showing that the ECG T-wave vector oscillation was enhanced during increased sympathetic activity and reduced following beta-adrenergic blockade.^2^ Recordings of ventricular APD (measured as ARIs) in patients showed that LF oscillations of APD were also increased following sympathetic provocation^1^ and decreased following beta-adrenergic blockade.^10^ In the study by Porter *et al*. sympathetic provocation was induced by the Valsalva manoeuvre during steady-state pacing in patients with an ICD. Recordings of left ventricular epicardial APD (ARI) showed an increase in LF power.^1^ In another study in patients undergoing routine electrophysiological procedures for supraventricular arrhythmia, unipolar electrograms were obtained from 10 right and 10 left ventricular endocardial sites during steady-state pacing. Acute beta-adrenergic blockade reduced LF oscillation of ARIs.^10^ Collectively, these studies support the contention that LF oscillations of the ECG T-wave vector reflect oscillations of ventricular APD and that the enhancement of the T-wave vector LF oscillations in response to enhanced sympathetic activity reflects the effect of phasic increases in sympathetic activity on ventricular APD.

## Electrophysiological mechanisms underlying low-frequency oscillations of ventricular action potential duration

**Figure 5:**
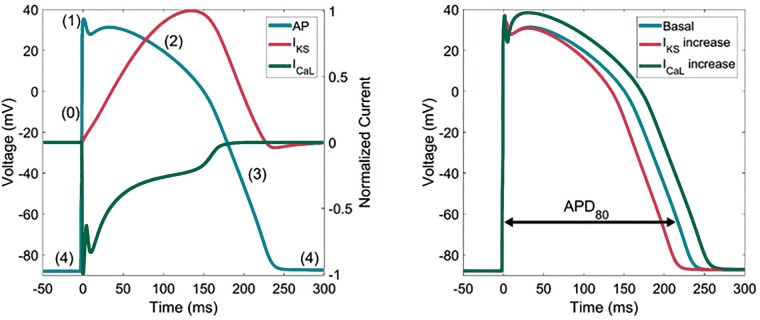
Left panel: Illustration of the timing of two currents thought to play a key role in generation oscillations of the ventricular action potential in humans. The inward L-type calcium current (I_CaL_) occurs early during phases 0, 1, and 2 and the outward potassium current (I_Ks_) occurs later during phases 2 and 3. Right panel: The overall effect of I_CaL_ is APD prolongation and the effect of I_Ks_ is APD shortening.

**Figure 6:**
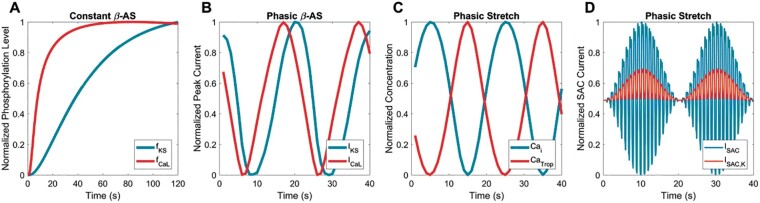
(*A*) Time course of normalized phosphorylation levels for the slow delayed rectifier potassium IKs current (fI_Ks_, blue) and L-type I_CaL_ current following constant beta-adrenergic stimulation. (*B*) Normalized peak current values for I_Ks_,(blue) and I_CaL_ current (red) following prolonged beta-adrenergic stimulation. (*C*) Systolic levels of free cytosolic calcium (Ca_i_, blue) and calcium bound to troponin (Ca _Trop_, red) following prolonged phasic and mechanical stretch. (*D*) Current through all stretch-activated channels (I_SAC_, blue) and through K+-selective stretch-activated channels (I_SACK_, red) following prolonged mechanical stretch.

What are the cellular mechanisms whereby phasic sympathetic activation generates an LF oscillatory pattern of APD? Beta-adrenergic stimulation initiates a signalling cascade in cardiac myocytes through G protein activation of adenyl cyclase which enhances cyclic AMP production and activation of protein kinase A. Protein kinase A phosphorylates multiple targets including regulating the L-type calcium current (I_CaL_) and the slowly activating delayed rectifier current (I_Ks_).^15^ Most studies examining the effect of beta-adrenergic stimulation on ventricular APD have reported a shortening.^16^ However, these observations have traditionally been made under steady state or near steady-state conditions. Studies in myocytes and *in silico* modelling have recently demonstrated a biphasic response of ventricular APD in the immediate few beats following abrupt beta-adrenergic stimulation.^17^^,^^18^ The application of Isoprenaline transiently prolonged APD for a few beats and then subsequently progressively shortened APD. This biphasic response was the result of a mismatch between the fast phosphorylation/dephosphorylation time constants of the L-type calcium current (I_CaL_) and the slower time constants of slow component of the delayed rectifier current (I_Ks_). The fast time constant of inward I_CaL_ current results in initial APD lengthening. After a few beats, outward I_Ks_ catches up, counterbalances I_CaL_, and induces APD shortening^17^^,^^18^ (*Figure 5*). Pueyo *et al.*^19^ used computational modelling to investigate the cellular mechanisms underlying LF oscillations of APD in response to phasic beta-adrenergic stimulation in the LF range. They found that I_CaL_/I_Ks_ mismatch following beta-adrenergic stimulation as described above could play a major role. For their computations, they simulated an oscillatory pattern of beta-adrenergic stimulation by consecutive 10 s on/off sequences of isoprenaline. A biphasic APD response was observed for each isoprenaline application, with initial transient APD prolongation accompanied by dominant I_CaL_ followed by APD shortening accompanied by dominant I_Ks_.^19^ Similarly, isoprenaline washout led to transient APD shortening followed by APD prolongation (*Figure 6A–C*).

## A contributory role of mechano-electric feedback to the generation of low-frequency oscillations of ventricular action potential duration

Beta-adrenergic stimulation increases cardiac contractile function by excitation contraction coupling^15^ with an increase in the force of contraction and muscle fibre excursion. These alterations in stress/strain patterns exert a feedback effect on the cardiac electrophysiology by a process known as mechano-electric feedback (MEF).^20–22^ Mechano-electric feedback is a complex process involving stretch-activated channels and calcium cycling mechanisms. Experimental work has shown that sympathetic provocation amplifies the effect of alterations in ventricular loading on the electrophysiology.^23^ Studies in patients during sympathetic provocation showed an increase in left ventricular contractility, measured as dp/dtmax, the first derivative of systolic pressure,^24^ and showed a correlation between the increase in LF power of APD and the LF power of dp/dt max. Pueyo and colleagues incorporated MEF in their modelling of mechanisms underlying LF APD oscillations, simulated as phasic LF changes in sarcomere length. Mechano-electric feedback exerted a synergistic effect with the beta-adrenergic-induced I_CaL_/I_Ks_ mismatch mechanism described above in the generation of LF oscillations of APD following enhanced sympathetic activity^19^ (*Figure 6D*).

Thus the foregoing provides a possible framework whereby phasic LF sympathetic stimulation may induce an oscillatory pattern of ventricular APD at the same frequency through cellular mechanisms which include a mismatch between the time constants of the L-type calcium current (I_CaL_) and the slow component of the delayed rectifier potassium current (I_Ks_) together with MEF.

## Oscillations of ventricular action potential duration and arrhythmogenesis: importance of disease conditions

**Figure 7:**
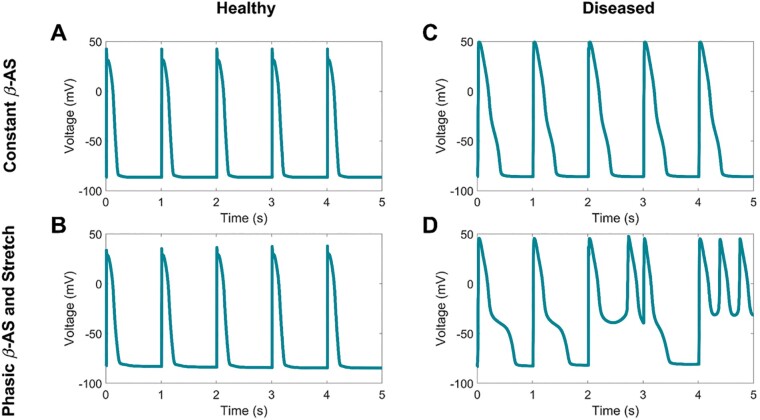
Simulated ventricular action potentials during beta-adrenergic stimulation (BAS). (*A*) Healthy myocardium during constant BAS, (*B*) Healthy myocardium during phasic BAS and stretch, (*C*) Myocardium with diseased conditions (simulated by addition of reduced repolarization reserve and calcium overload) during constant BAS, (*D*) Diseased conditions during phasic BAS and stretch. In C early afterdepolarizations are seen but no arrhythmia occurred. In D early afterdepolarizations and triggered activity are evident (see text).

The autonomic nervous system, particularly enhanced sympathetic activity, has long been known to play an important role in arrhythmogenesis.^25^^,^^26^ The majority of studies investigating mechanisms have mainly Focused on steady-state conditions and until recently relatively little attention had been given to transient or oscillatory dynamics. As described above Liu *et al.*^17^ showed in genetically engineered rabbit cardiac myocytes that the mismatch between the faster phosphorylation/dephosphorylation kinetics of I_CaL_ and the slower I_Ks_ kinetics following isoprenaline could result in a window after about 5–10 beats when I_CaL_ could be reactivated and generate EADs and triggered activity. Pueyo *et al* simulated consecutive 10 or 20 s cycles of beta-adrenergic stimulation in the presence of disease conditions by incorporating calcium overload and reduced repolarization reserve, modelled as reduced rapid delayed rectifier potassium current (I_Kr_) and reduced slow component of the delayed rectifier potassium current (I_Ks_). They found that the LF power of APD oscillations was substantially increased and early afterdepolarizations and runs of triggered activity were observed^19^ (*Figure 7*). In an established A-V block dog model, ventricular APD was measured using monophasic action potentials.^13^ Low-frequency oscillations were present under control conditions in sinus rhythm and the LF power increased following acute A-V block, and increased further in chronic A-V block conditions (2 weeks later) attributed to the effect of ventricular remodelling. Inducibility of Torsades de Pointes with dofetilide (I_Kr_ blocker) showed that LF power of APD was larger in inducible chronic A-V block dogs.^13^ Thus both modelling and experimental work provide a possible mechanistic basis for a role of oscillatory repolarization dynamics in generating afterdepolarization and highlight the importance of the presence of disease/remodelling In facilitating arrhythmogenesis.

## Interaction between low-frequency oscillation of ventricular action potential duration and proarrhythmic beat-to-beat variability of repolarization

Beat-to-beat variability of ventricular repolarization (BVR) has been shown to be proarrhythmic in a wide range of experimental models and humans particularly when enhanced in response to beta-adrenergic stimulation.^12^^,^^27–30^ In a recent study in patients with an ICD,^11^ beat-to-beat variability of left ventricular epicardial APD (measured as ARI during RV pacing) was shown to increase following sympathetic provocation (Valsalva). This effect was almost entirely eliminated by a beta-adrenergic blocking agent. An interactive effect has been demonstrated between LF oscillation of APD and BVR.^11^ The mechanism for this interaction at the cellular level was investigated using computer simulation. The major ionic contributors to concomitant variations in LF oscillation of APD and BVR were the magnitudes of I_Kr_, I_CaL_, and the inward rectifier potassium current (I_K1_).^31^ The same three ionic currents were found to explain the development of proarrhthmic events in the form of afterdepolarizations and runs of spontaneous beats in response to enhanced sympathetic activity.^31^

## Theoretical considerations

In the foregoing, we have reviewed evidence supporting the contention that LF oscillations of ventricular repolarization are related to the effect of rhythmic sympathetic nerve activity on APD. However, the PRD is an electrocardiographic phenomenon and could also be influenced by structural changes and functional properties of the myocardium. For example, it has been suggested that the different intrinsic properties of myocardial cells across the ventricular wall observed in single cells may play a role.^2^ However, in the whole heart where cells are electrically and mechanically coupled differences in APD between cells may be markedly attenuated by electrotonic interaction.^32^^,^^33^ In diseased hearts, the presence of structural changes such as scar and fibrosis can impact on repolarization. Therefore, in these hearts, phasic sympathetically mediated changes in APD might be expected to induce phasic increases in dispersion of repolarization and contribute to the PRD.

## Clinical implications

Low-frequency oscillatory behaviour of ventricular repolarization may provide a novel approach to both risk stratification and mechanisms of arrhythmogenesis. There is urgent need to improve risk stratification for the use of ICD devices for the prevention of ventricular arrhythmia and sudden cardiac death. Current guidelines from the American Heart Association, American College of Cardiology, and the European Cardiac Society recommend prophyllactic ICD implantation in patients with ischaemic and non-ischaemic cardiomyopathy with ejection fraction below 35%.^34^^,^^35^ Although ICD implantation has proved to be highly effective, less than 1 in 10 of the implanted devices are actually needed. Hence a large number of patients are unnecessarily exposed to side effects such as infection and inappropriate shocks, and needlessly contribute to the escalating cost estimated at in excess of 2 billion euros per annum in Europe alone. In ICD patients, PRD <7.5 deg was associated with only a 31% reduction in mortality by the device compared to a 75% reduction in patients with PRD >7.5 deg. Numbers need to treat to prevent one death were reduced from 18.3 in patients with PRD <7.5 deg compared to 3.1 in patients with PRD >7.5 deg. Periodic repolarization dynamics is a dynamic measure operating over a time frame of seconds in contrast to a number of other risk markers which measure static or near static properties.^36^ Several dynamic tests have proven value as risk predictors of sudden cardiac death such as baroreceptor sensitivity,^37^ heart rate turbulence,^38^ deceleration capacity,^39^ microvolt T-wave alternans,^40^ and tests of RR interval dynamicity.^41^ The mechanistic link between each of these risk predictors and arrhythmia is highly complex but a main focus centres round separating the balance between sympathetic and parasympathetic activity, whereas PRD may relate more to the electrophysiological time constants that govern the repolarization process. The potential value of incorporating the dimension of time into risk stratification protocols has been suggested particularly in regard to tests incorporating autonomic function.^36^^,^^42^ Future work might focus of elucidating the physiology and electrophysiology of these dynamic markers in combination. This approach may be beneficial not only for risk stratification strategies but also for the development of anti-arrhythmic drug therapy and device therapy.

## Conclusions

The powerful role of LF oscillatory dynamics of ventricular repolarization in the prediction of ventricular arrhythmia and sudden cardiac death is now firmly established.^2–4^ It was proposed that these oscillations reflect oscillations of ventricular APD at the sympathetic nerve frequency. There is now a substantial body of evidence to support this contention and to provide a framework for underlying mechanisms. Specifically, the demonstration of the existence of oscillatory behaviour of ventricular APD in humans; its amplification by sympathetic activity and reduction by beta-adrenergic blockade similar to LF T-wave vector oscillations; the demonstration of possible cellular mechanisms including mismatch of the phosphorylation kinetics of ICa and I_Ks_ in response to beta-adrenergic stimulation together with MEF; the generation of afterdepolarizations and triggered activity in the presences of disease conditions and interaction between LF oscillations of ventricular APD with other proarrhythmic mechanisms.

### Funding

E.P. and D.A.S.-P. were supported by the Eur Research Council under grant agreement ERC-StG 638284 by Ministerio de Ciencia e Innovacion (Spain) through project PID 2019-105674RB-100 and by European Social Fund (EU) and Aragon government through BSICoS group (T39/2OR) and project LMP 124–18. Computations were performed by the ICTS NANBIOSIS (HPC) Unit at University of Zaragoza.

**Conflict of interest:** none declared.
